# Light-induced Amaurosis: A Rare Manifestation of Internal Carotid Artery Stenosis

**DOI:** 10.7759/cureus.5817

**Published:** 2019-10-01

**Authors:** Ru Jin Eugene Ting, Michelle Hui, Catherine Thoo, Minas T Coroneo, Ian C Francis

**Affiliations:** 1 Department of Ophthalmology, The University of Sydney, Sydney, AUS; 2 Department of Ophthalmology, Prince of Wales Hospital, Sydney, AUS; 3 Department of Vascular Surgery, Royal Hobart Hospital, Tasmania, AUS

**Keywords:** light-induced amaurosis, hemeralopia, carotid stenosis, amaurosis fugax, transient ischaemic attack, stroke

## Abstract

Light-induced amaurosis (LIA) is a rare presentation of internal carotid artery (ICA) stenosis. This report documents a 74-year-old Caucasian male who presented with profound right monocular vision loss, occurring on every occasion upon entering brightly lit environments. This was managed successfully with a right carotid endarterectomy.

This case is presented to highlight the recognition and understanding of LIA and its importance for preservation of vision and prevention of ICA-related stroke.

## Introduction

Atherosclerotic disease of the internal carotid artery (ICA) can be categorised as either symptomatic [1-5] or asymptomatic [6]. Symptomatic patients may present with vision loss secondary to amaurosis fugax [2], a transient ischaemic attack or a cerebrovascular event. However, light-induced amaurosis (LIA) can also rarely occur. LIA is manifested when a patient moves into a bright environment, and it is thought to be a haemodynamic disorder rather than an embolic phenomenon [5]. There is a dearth of reported cases of ICA stenosis resulting in LIA [1-5]. The term LIA is distinct from hemeralopia, which is poor vision in bright light. Interestingly, in terms of terminology, the French literature does not adequately differentiate hemeralopia as occurring in either dark or bright conditions [7].

Although there are other causes for LIA, including hereditary retinal dystrophies such as cone dystrophies, every patient presenting with new LIA, particularly with a carotid bruit [8], should be investigated with carotid studies with reasonable alacrity. This is because significant carotid stenosis may underlie LIA, and if managed adequately, may preserve vision, prevent stroke, and possibly prevent death [9].

## Case presentation

A 74-year-old Caucasian man with known cataracts presented to an ophthalmic surgeon with a one-month history of right vision loss when exposed to a bright environment. On detailed questioning, he reported that he ‘went completely blind’ in his right eye, and in fact described a ‘white out’ every time he went outside during the day. This would persist for several minutes, but the patient reported no other focal neurological symptoms. His past medical history included longstanding right carotid stenosis, ischaemic heart disease, implantation of a pacemaker/defibrillator, Type 2 diabetes mellitus, and polymyalgia rheumatica. He also had atrial fibrillation for which he was treated with digoxin and amiodarone and was anticoagulated with warfarin. He was an ex-smoker with a 32-pack-year history until the age of 50.

Examination revealed bilateral carotid bruits, but normal heart sounds. There were no focal neurological findings and no clinical features of temporal arteritis [10,11]. He had bilateral nuclear sclerotic, cortical, and posterior subcapsular cataracts. Due to the presence of carotid bruits and vision loss, he was referred promptly for carotid duplex studies and vascular surgical consultation.

**Figure 1:**
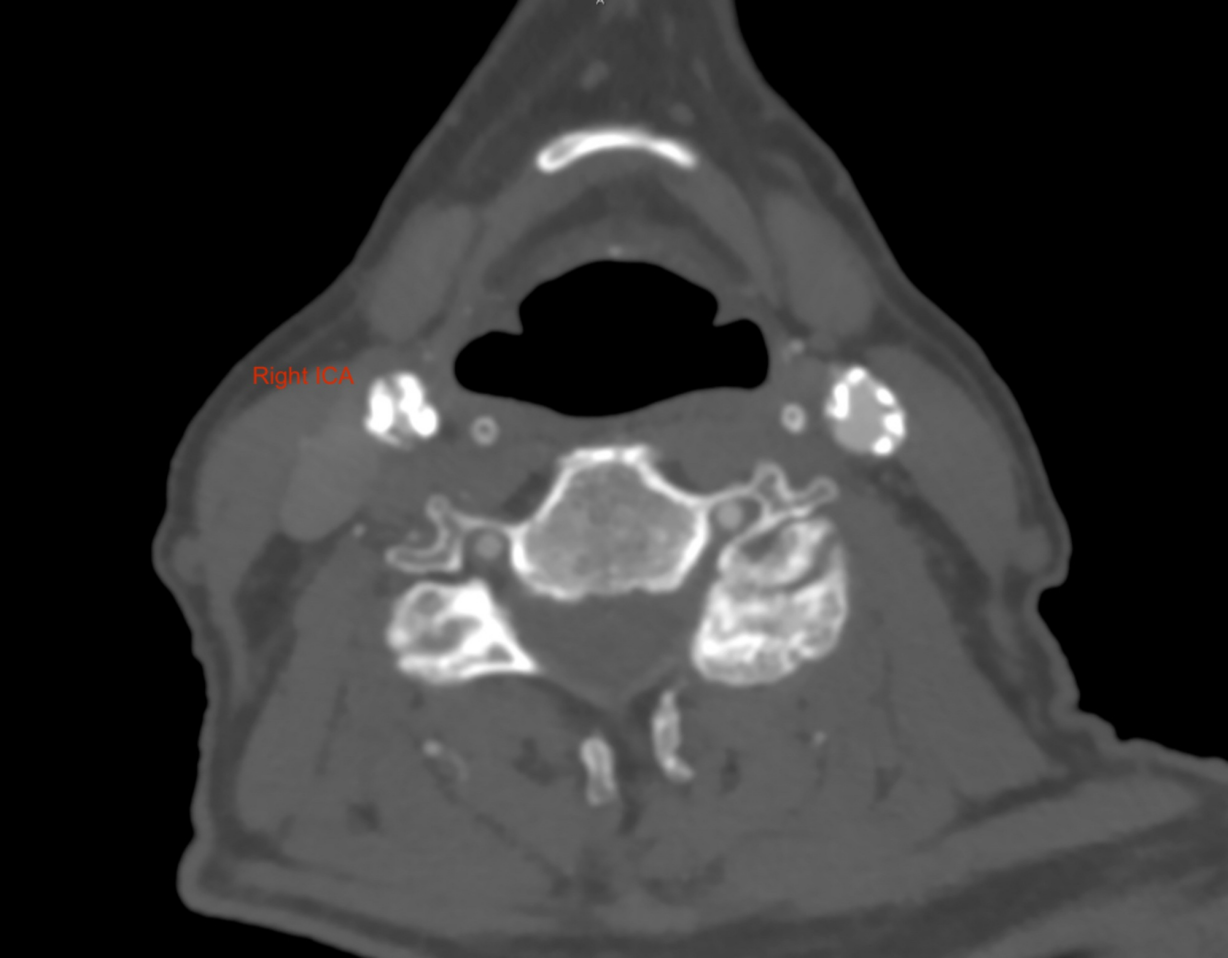
Computed tomography-angiogram (CT-A), axial section, demonstrating high grade stenosis of the right internal carotid artery (ICA).

Carotid duplex doppler ultrasonography suggested a moderate (50%-69%) stenosis of the right ICA. The left ICA had generally raised velocities but no specific lesions were seen. Computed tomography-angiography (CT-A) demonstrated a high grade (80%-99%) stenosis of the right ICA (Figure 1).

**Figure 2:**
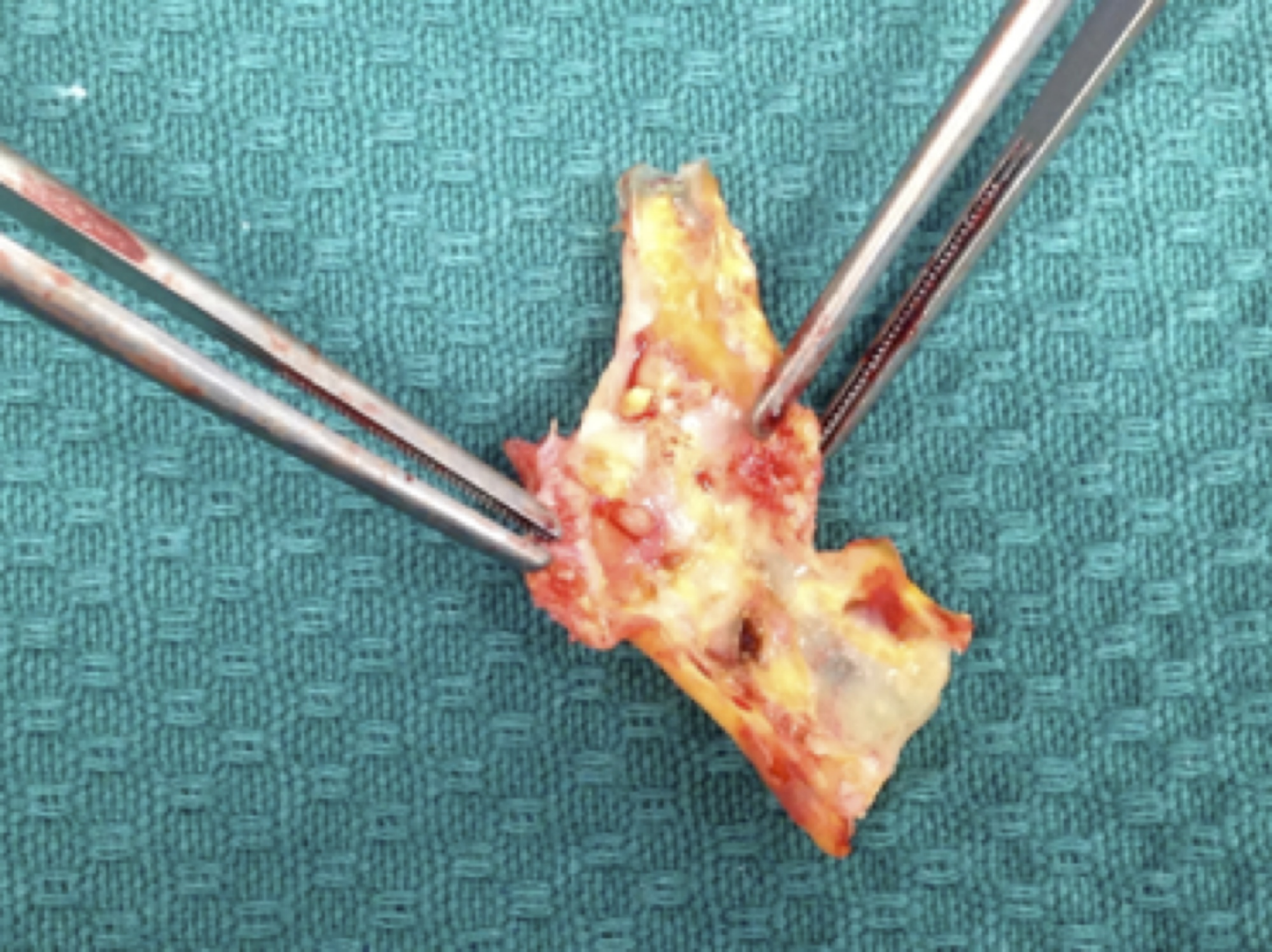
The haemorrhagic, ulcerated plaque that caused very high grade stenosis in the right internal carotid artery (ICA). This photograph was taken immediately after surgery.

Within 36 hours, the patient underwent a right carotid endarterectomy (CEA) and patch, performed under infiltration local anaesthesia with sedation. Shunting was not required as the patient remained neurologically intact during carotid artery clamping. Intraoperatively, a heterogeneous haemorrhagic ulcerated plaque (Figure 2), causing the high grade stenosis, was identified. CEA was performed with good endpoints, and a Braun® Patch (B. Braun, Melsungen, AG) was sewn onto the ICA with 6/0 prolene. Postoperatively, the patient was monitored overnight in the intensive care unit (ICU). There were no new neurological symptoms, and he remained haemodynamically stable. He was discharged home the following day.

Initially, there was no change in his symptomatology. However, after three weeks he reported a sudden marked and exponential improvement in his visual function, with no further episodes of LIA. At review six weeks postoperatively, his neurological status remained normal, and carotid duplex ultrasonography demonstrated the right ICA to be widely patent.

## Discussion

This case documents a patient who developed recurrent episodes of 'white out' of the vision in his right eye, (LIA), due to internal carotid artery stenosis, successfully managed with CEA.

The most common visual presentation in patients with unilateral ICA stenosis is amaurosis fugax, and it occurs in 6.6% of symptomatic cases [1]. This is characterized by sudden, transient, unilateral vision loss, which may be complete, partial, or related to a specific part of the visual field. Amaurosis fugax is unrelated to exposure to bright light and is considered to be a result of microembolisation from an ipsilateral ICA stenosis [4]. LIA is a distinct entity that occurs when patients move into a brightly lit environment, and it is thought to be related to the haemodynamics of carotid blood flow [5]. Although rare, it is important because its recognition is necessary for timely vascular intervention due to the major risk for stroke.

Late last century, the group led by Professor Thomas P. Kearns, who was at that time the Professor of Ophthalmology at the Mayo Medical School, Rochester and former President of the American Academy of Ophthalmology, reported five cases with LIA related to ICA stenosis [5].

The first case of Kearns et al. typifies the lateralising value of LIA to the side of the carotid stenosis [5]. In contrast, in their third case, the patient had right LIA with an occluded right ICA, indicating that the aetiology of the LIA was not due to ipsilateral ICA stenosis [5]. Thus, the side of the LIA, let alone the amaurosis fugax, may not be localising in terms of the involved ICA. Their second case presented with ‘white haziness’ of vision as a manifestation of LIA, symptomatology similar to that of our case [5].

The unifying symptomatology of the case series by Kearns et al. is visual loss related either to posture, exposure to bright light, or both [5]. While all patients in this case series had LIA, adoption of the sitting posture from the supine posture led to amaurosis fugax in three of the cases, indicating a haemodynamic rather than an embolic disorder [5].

Under normal physiologic conditions, exposure of the retina to light results in bleaching of photoreceptor pigments [5]. The resynthesis of these pigments determines the rapidity of visual adaptation to light and requires high energy-utilising metabolic processes [5]. It is believed that in the setting of ICA stenosis and subsequent retinal hypoperfusion, the increase in retinal metabolism demanded by the exposure to light cannot be sustained. This results in a specific maladaptation to bright light, manifesting as LIA [5,12].

Other causes of LIA and altered light-adaptation may include disturbances in the phototransduction cascade due to retinal disease, although this is uncommon. Mutations in the RGS9-1 or R9AP genes have been shown to affect light adaptation. Retinal disorders such as cone dystrophy and albinism are known to produce hemeralopia [13]. None of these conditions were relevant to our patient.

Nevertheless, many common ophthalmological conditions may present with glare or reduced vision on exposure to bright light. These include cataracts, diffractive and refractive problems (such as corneal oedema, lens subluxation, and astigmatism), anterior uveitis, and in eyes with large/distorted pupils.

## Conclusions

Patients presenting with LIA should be thoroughly assessed ophthalmologically and neuro-ophthalmologically, and they should be referred for carotid studies as necessary. Depending on the outcome, vascular surgical intervention may be beneficial.

## References

[1] Hoya K, Morikawa E, Tamura A, Saito I (2008). Common carotid artery stenosis and amaurosis fugax. J Stroke Cerebrovasc Dis.

[2] Kaiboriboon K, Piriyawat P, Selhorst JB (2001). Light-induced amaurosis fugax. Am J Ophthalmol.

[3] Roberts DK, Sears JM (1992). Light-induced amaurosis associated with carotid occlusive disease. Optom Vis Sci.

[4] Ramirez-Lassepas M, Sandk BA, Burton RC (1973). Clinical indicators of extracranial carotid artery disease in patients with transient symptoms. Stroke.

[5] Furlan AJ, Whisnant JP, Kearns TP (1979). Unilateral visual loss in bright light. An unusual symptom of carotid artery occlusive disease. Arch Neurol.

[6] Ignat’ev IM (2012). Asymptomatic stenoses of carotid arteries. [Article in English, Russian]. Angiol Sosud Khir.

[7] N Ohba, A Ohba (2006). Nyctalopia and hemeralopia: the current usage trend in the literature. Br J Ophthalmol.

[8] McColgan P, Bentley P, McCarron M, Sharma P (2012). Evaluation of the clinical utility of a carotid bruit. QJM.

[9] Flaherty ML, Kissela B, Khoury JC (2013). Carotid artery stenosis as a cause of stroke. Neuroepidemiology.

[10] Hollenhorst RW, Kublin JG, Millikan CH (1963). Ophthalmodynamometry in the diagnosis of intracerebral orthostatic hypotension. Proc-Staff Meet Mayo Clin.

[11] George A, Lim NS, Jain NS (2016). “Diagnositc algorithm for patients with suspected giant cell arteritis” useful but no substitute for thorough histopathology. J Neuroophthalmol.

[12] Ross Russell RW, Page NGR (1983). Critical perfusion of brain and retina. Brain.

[13] Nishiguchi KM, Sandberg MA, Kooijman AC (2004). Defects in RGS9 or its anchor protein R9AP in patients with slow photoreceptor deactivation. Nature.

